# *Agrobacterium*-mediated transformation of safflower and the efficient recovery of transgenic plants via grafting

**DOI:** 10.1186/1746-4811-7-12

**Published:** 2011-05-20

**Authors:** Srinivas Belide, Luch Hac, Surinder P Singh, Allan G Green, Craig C Wood

**Affiliations:** 1 CSIRO Plant Industry, PO Box 1600, Canberra, ACT 2601, Australia; 2 Department of Biotechnology, Sreenidhi Institute of Science and Technology, Yamnampet, Ghatkesar, Hyderabad-501301, AP, India

## Abstract

**Background:**

Safflower *(Carthamus tinctorius L.) *is a difficult crop to genetically transform being susceptible to hyperhydration and poor *in vitro *root formation. In addition to traditional uses safflower has recently emerged as a broadacre platform for the production of transgenic products including modified oils and pharmaceutically active proteins. Despite commercial activities based on the genetic modification of safflower, there is no method available in the public domain describing the transformation of safflower that generates transformed T_1 _progeny.

**Results:**

An efficient and reproducible protocol has been developed with a transformation efficiency of 4.8% and 3.1% for S-317 (high oleic acid content) and WT (high linoleic acid content) genotypes respectively. An improved safflower transformation T-DNA vector was developed, including a secreted *GFP *to allow non-destructive assessment of transgenic shoots. Hyperhydration and necrosis of *Agrobacterium*-infected cotyledons was effectively controlled by using iota-carrageenan, L-cysteine and ascorbic acid. To overcome poor *in vitro *root formation for the first time a grafting method was developed for safflower in which ~50% of transgenic shoots develop into mature plants bearing viable transgenic T_1 _seed. The integration and expression of secreted *GFP *and hygromycin genes were confirmed by PCR, Southern and Western blot analysis. Southern blot analysis in nine independent lines indicated that 1-7 transgenes were inserted per line and T_1 _progeny displayed Mendelian inheritance.

**Conclusions:**

This protocol demonstrates significant improvements in both the efficiency and ease of use over existing safflower transformation protocols. This is the first complete method of genetic transformation of safflower that generates stably-transformed plants and progeny, allowing this crop to benefit from modern molecular applications.

## Background

Safflower is a versatile crop with several desirable traits, multiple applications and is well adapted to semi-arid conditions in the tropics and subtropics. Safflower is grown for its edible oil (high oleic and high linoleic varieties), high-protein seed cake, animal meal, bird seed and for traditional medicine [1,2]. Apart from these traditional uses safflower has recently emerged as a broadacre platform for the production of transgenic products, including modified oils such as gamma-linolenic acid [3] and pharmaceutically active proteins including human insulin and apolipoprotein [4,5]. Safflower has become an industrial crop production platform based on low out-crossing and weediness habits, a different appearance from other oilseed crops such as canola and excellent agronomic traits such as taproot architecture that accesses sub-soil water reserves [6].

Despite commercial activities based on the genetic modification of safflower, there is no method available in the public domain describing the generation and analysis of transgenic T_1 _progeny. The lack of a reliable regeneration of transgenic T_1 _progeny in safflower not only limits its potential as an industrial crop production platform but also the application of modern molecular techniques to investigate and improve this economically-important plant. Undoubtedly safflower is a difficult crop to genetically engineer, and a large body of literature describes a series of limitations in tissue culture approaches [7-9]. Firstly, under *in vitro *conditions safflower shoots are susceptible to hyperhydration, a physiological disorder with aberrant morphology, characterized by swollen translucent leaves and brittle stems. Hyperhydration is likely caused by two important factors - the gelling agent of the media and high humidity in culture vessels. The risk of hyperhydration increases in post-transformation selection because of prolonged culture on antibiotic selection media. Therefore the use of antibiotic selection agents in safflower transformation methods presents a dilemma: antibiotic selection agents are required to reduce the number of 'false positives' yet also contribute to the occurrence of hyperhydration. One possible alternative to antibiotic selection agents in the culture media is the use of fluorescent proteins, such as *GFP*, as a visual marker to aid in the identification of transgenic shoots [10].

A second major limitation in safflower transformation is the poor formation of roots from transformed shoots, a vital developmental step towards the generation of mature T_0 _plants and seeds. *In vitro *induced roots are often very weak and frequently fail to survive the transfer from tissue culture media to soil [11]. Faced with the dual issues of hyperhydration and poor *in vitro *root formation an alternative method [12] of safflower transformation was developed, called *in planta *transformation. In this approach the embryo of the young seedling is pierced and co-cultivated with an *Agrobacterium *harboring the T-DNA vector. Over time the embryo develops into mature safflower plants containing segments of transformed sectors, so called chimeric T_0 _plants. Although this approach overcomes both hyperhydration and *in vitro *root formation there is little convincing evidence that this *in planta *method reliably generated transformed T_0 _plants or, most critically, transformed T_1 _progeny. Poor *in vitro *root formation was also reported in other dicot crops and has been addressed using grafting techniques to enhance plant recovery from somatic embryos and genetically transformed shoots [13-16] including sunflower [17,18].

Here we report the development of a protocol for the reliable production of genetically-transformed safflower. We used antioxidants (L-cysteine, ascorbic acid) during co-cultivation and selection to reduce necrosis, iota-carrageenan with modified gelling strength to control the hyperhydration and a non-cytotoxic *GFP *visual marker system to identify GM-shoots. GM-shoots were grafted, using a novel *in vivo *grafting technique, to recover and establish mature transgenic safflower plants. Various molecular techniques demonstrate that this method generated mature T_0 _plants and viable stably-transformed T_1 _seed. This complete report of a reliable method for the genetic transformation of safflower will help this important crop to benefit from advances in plant biotechnology.

## Results and Discussion

### 1. Construction and testing of a binary vector suitable for safflower transformation

**Figure 1:**
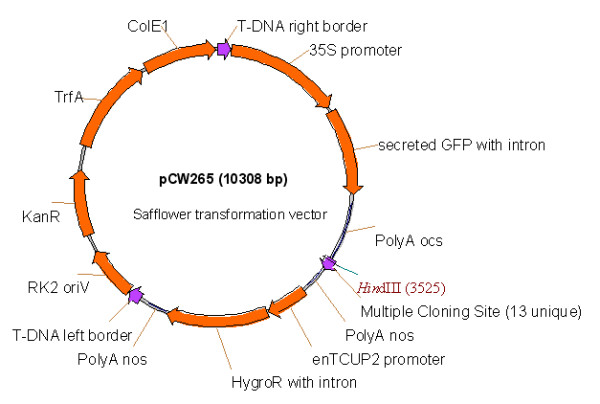
**Schematic representation of the T-DNA region of the T-DNA binary vector developed for the transformation of safflower**. Secretion of the *GFP *into the apoplast is required for healthy safflower development. Both the *GFP *and hygromycin CDS regions include a catalase-1 intron to abolish expression in *Agrobacterium*. The multiple cloning site (MCS) includes unique sequences for digestion by *BstEII, SbfI, HindIII, XbaI, XmaI, SmaI, SalI, ClaI, EcoICRI, SacI, Acc65I, Spe1 *and *EcoRV*. A complete explanation of the features of the vector backbone are found in Coutu *et al.*, [19] and in the text.

The binary vector pORE3 [19] was modified so that the kanamycin resistance gene, *npt-II*, was replaced with the hygromycin resistance gene, *hph*, from the pVEC8 vector series [20], creating pORE6. This version of *hph *contains a catalase-1 intron rendering the gene inactive in *Agrobacterium*. In our hands this intron-interrupted version of *hph *greatly reduced bacterial overgrowth in post-transformation selection and regeneration experiments, similar to previously reported results [20]. Preliminary experiments in safflower transformation experiments indicated that a GFP located within the cytoplasm was cytotoxic (Additional file 1), an effect that was previously overcome by targeting the GFP to the endoplasmic reticulum of the cell [10]. To overcome cytotoxicity effects a GFP located to the apoplast was designed following the protocol [21] where the secretion peptide of conglycinin was translationally-fused to the N-terminal of GFP and a four glycine amino acid stretch was fused to the C-terminal of GFP. The secreted GFP was placed under the control of the 35S promoter, generating the plant binary vector pCW265 (Figure 1). Safflower was transformed with pCW265 and the location of this secreted GFP was confirmed as apoplastic (Additional file 2). pCW265 contains a multiple cloning site region to allow convenient ligation of genes of pathways of interest for expression in safflower.

### 2. Optimized *Agrobacterium *pre-treatments and cell density for T-DNA transfer into safflower

**Figure 2:**
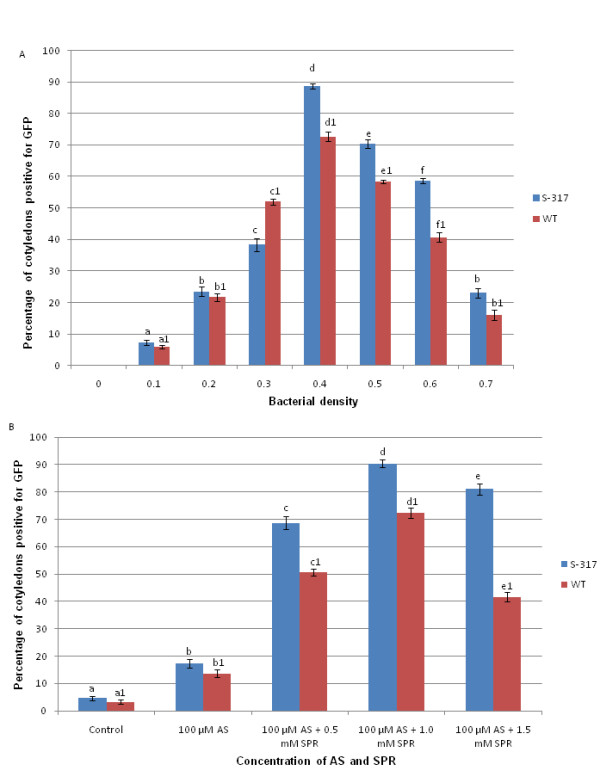
**Optimization of *Agrobacterium *infection pre-treatments for T-DNA transfer into safflower cotyledon explants**. (A) Effect of bacterial density on transient expression of *GFP *in cotyledons of S-317 and WT. (B) Acetosyringone and spermidine concentration in inoculation media (Winnans AB salts) and its effect on *GFP *expression two weeks after *Agrobacterium *co-cultivation (OD600 = 0.4). Each value is the mean ± SE of three independent experiments, each with 30 cotyledons. Significant differences between treatments are indicated by different letters.

Preliminary transformation experiments with pCW265 determined that the optimal cell density of *Agrobacterium *for infection was OD600 = 0.4 for both genotypes of safflower (Figure 2A) and this density was used throughout all subsequent experiments. Acetosyringone (AS) [22] and spermidine (SPR) [23] are potent inducers of virulence (*vir*) genes of *Agrobacterium *and are used in many plant species for successful transformation. The effect of AS and SPR on T-DNA delivery into safflower cotyledons was tested (Figure 2B) by scoring the percentage of calli with a *GFP *fluorescence signal after co-cultivation with *Agrobacterium *harbouring pCW265 and two weeks culture on S2 selection media (Table 1). A combination of 100 μM of AS and 1.0 mM of SPR was found to be optimal for both genotypes, with as many as 70-90% of explants scoring positive for secreted *GFP *expression. A further increase in SPR concentration from 1 mM to 1.5 mM resulted in reduced T-DNA delivery in both genotypes. Our results suggest that the combination of AS and SPR as pretreatments for *Agrobacterium *improved the transformation efficiency in safflower.

**Table 1:** Composition of media for transformation of safflower from cotyledon explants.

Component	S_1 _Co-cultivation		S_2 _Callus induction		S_3 _Shoot initiation		S_4 _Shoot out-growth		S_5 _Shoot elongation	
	
	S-317	WT	S-317	WT	S-317	WT	S-317	WT	S-317	WT
Media	MS[33]	MS	MS	MS	MS	MS	MS	MS	MS	MS
*Growth regulators*										
TDZ	-	1	-	1	-	-	-	-	-	-
NAA	0.1	0.1	0.1	0.1	-	-	-	-	-	-
BA	1	-	1	-	1	-	-	-	-	-
KN	-	-	-	-	-	1	-	-	-	-
2iP	-	-	-	-	-	-	-	0.5	-	0.5
GA	-	-	-	-	-	-	-	-	0.1	0.1
*Antioxidants*										
L-cysteine	50	50	-	-	-	-	-	-	-	-
Ascorbic acid	15	15	-	-	-	-	-	-	-	-
PVP	-	-	-	-	-	-	-	-	500	500
*Antibiotics*										
Hygromycin	-	-	20	20	20	20	18	18	-	-
Cefotaxime	-	-	250	250	250	250	250	250	250	250
Timentin *Others*	-	-	50	50	50	50	50	50	50	50
*Iota-*carrageenan	-	-	1500	1500	1500	1500	1500	1500	-	-
AgNo_3_	-	-	3	3	3	3	1.5	1.5	1.5	1.5
Sucrose	30000	30000	30000	30000	30000	30000	20000	20000	20000	20000
Agar	9000	9000	9000	9000	9000	9000	-	-	-	-
Phytagel	-	-	-	-	-	-	5000	5000	5000	5000

Amounts indicate the mg l^-1 ^of each component. S-317 and WT refer to high oleic acid content seeds and high linoleic acid content seeds, respectively.

### 3. Necrosis is significantly reduced by antioxidants L-cysteine and ascorbic acid

**Figure 3:**
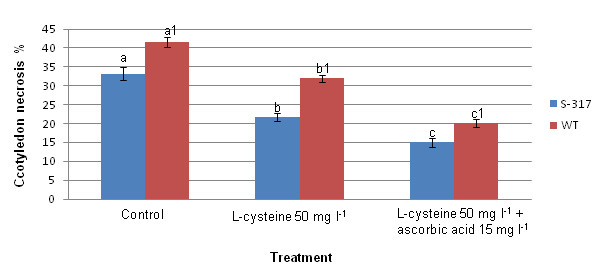
**The effect of L-cysteine and ascorbic acid on cotyledon necrosis**. Each value is the mean ± SE of three independent experiments, each with 30 cotyledons. Significant differences between treatments are indicated by different letters in each group of bars.

It has been demonstrated previously that co-cultivation of safflower explants with *Agrobacterium *decreases regeneration frequency compared with non-transformed controls and the addition of AS further reduced safflower regeneration because of the increased bacterial infectivity and resulting hypersensitive response [8]. Necrosis at the proximal end of the cotyledons following co-cultivation with *Agrobacterium *was observed in the present study. As poor regeneration from transformed cells ultimately limits the production of transgenic plants, we focused on improving the regeneration by reducing necrosis of the cotyledon through the addition of antioxidants L-cysteine and ascorbic acid (Figure 3). The best concentration of antioxidants was 15 mg l^-1 ^ascorbic acid and 50 mg l^-1 ^L-cysteine which reduced necrosis of cotyledons by half in the two genotypes tested. The antioxidants may help the post-infection survival of competent cells in and around the proximal end of the cotyledon, thereby increasing the number of transformed cells surviving to form calli and shoots. The combination of L-cysteine and ascorbic acid in co-cultivation medium may be also minimize cell death caused by the hypersensitive response of the cotyledon cells due to *Agrobacterium *infection.

### 4. Plant regeneration without hyperhydration

Safflower cotyledon explants were chosen as the target tissue for genetic modification. In the WT genotype MS media with 1 mg l^-1 ^TDZ and 0.1 mg l^-1 ^NAA was found optimal for induction multiple shoots with little or no callus formation. In contrast, the S-317 genotype required MS media with 1 mg l^-1 ^BA and 0.1 mg l^-1 ^NAA for the induction of multiple shoots. With the tested combination of hormones the frequency of shoot regeneration ranged from 85-90% with as many as 8-10 shoots emerging from each cotyledon explant. Such high efficiencies of shoot regeneration with similar phytohormone regimes has been reported [24].

**Figure 4:**
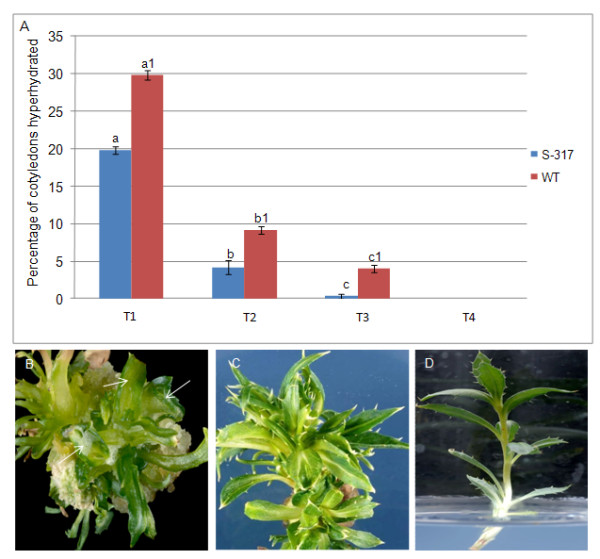
**The effect of iota- carrageenan and modified agar concentration on hyperhydration of safflower shoots**. (A) Media composition and percentage of cotyledons hyperhydrated after co-cultivation with *Agrobacterium *and 3-4 weeks on selection medium (Table 1). T1 = S2 + 8 g l^-1 ^Agar; T2= S2+9 g l^-1 ^Agar; T3 = S2 + 9 g l^-1 ^Agar +1.5 g l^-1 ^carrageenan; T4 = S2 + 9 g l^-1 ^Agar +2.5 g l^-1 ^carrageenan. Each value is the mean ± SE of three independent experiments, each with 30 cotyledons. Significant differences between media composition are indicated by different letters in each group of bars. (B) An example of hyperhydrated shoots grown on MS media with 8 g l^-1 ^agar and the arrow indicates the abnormal appearance of leaf. (C) Proliferation of normal and healthy shoots on MS media with 9.0 g l^-1 ^agar and 1.5 g l^-1 ^iota-carrageenan; (Note the sharp margins on leaves compared to the image in panel B). (D) A single healthy shoot on elongation media, S5, and ready for grafting.

A major problem faced both in this and other safflower transformation studies is the hyperhydration of transgenic shoots which result in the loss of a large proportion of transgenic shoots [7,8,25]. Different concentrations of agar and iota-carrageenan were tested for their ability to reduce symptoms of hyperhydricity in two safflower genotypes that had previously been inoculated with *Agrobacterium *(Figure 4A). Increasing the agar concentration from 8 g l^-1 ^to 9 g l^-1 ^reduced the incidence of hyperhydration from 20% to 4% in S-317 and from 30% to 9% in WT. When iota-carrageenan was added at 1.5 g l^-1 ^to MS media with 9 g l^-1 ^agar, hyperhydration was further reduced to 0.33% and 4.0% in S-317 and WT cotyledons, respectively. Further increases in the concentration of iota-carrageenan from 1.5 g l^-1 ^to 2.5 g l^-1 ^abolished hyperhydration but also severely impaired the growth (data not shown). The use of 9 g l^-1^agar and 1.5 g l^-1 ^iota-carrageenan was effective in controlling the hyperhydration in both genotypes of safflower without inhibiting growth and shoot proliferation, and these conditions were maintained up to the shoot out-growth stage (S4 media, Table 1). Figure 4B-D shows an example of a hyperhydrated shoot, regenerated on MS media with 8 g l^-1 ^agar, and a healthy shoot, regenerated on modified MS media containing the optimised agar and iota-carrageenan concentrations. A similar reduction in hyperhydration was reported in *Eucalyptus *shoots using iota-carrageenan [26]. The mechanism by which iota-carrageenan reduces hyperhydration is unclear but may be related to polysaccharides, such as galactans, as part of their structure that may play a role in reducing osmotic stress in the cell wall.

### 5. Visual screening of transgenic shoots using a secreted *GFP*

**Figure 5:**
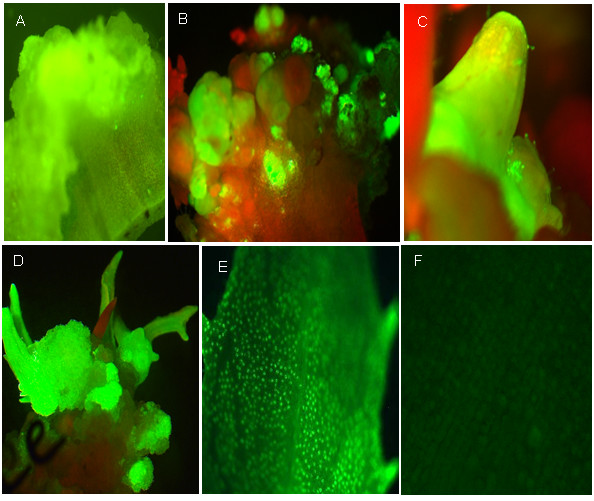
**The use of a *GFP *visual marker to rapidly identify transgenic callus and shoots of safflower**. (A) *GFP *expression in proximal end of the cotyledon two weeks after *Agrobacterium *infection. (B) Shoot bud initiation from the proximal end of cotyledon in the presence of 18 mg l^-1 ^hygromycin after four weeks of co-cultivation (C-D) Transformed shoots regenerating on selection media in presence of 18 mg l^-1 ^hygromycin (E) Visualization of *GFP *signals in guard cells of a transgenic leaf. (F) A non-transformed leaf/shoot showing no *GFP *signal and low background fluorescence.

In order to reduce the prolonged culture on antibiotic selection media and to identify transformed shoots at early stages of regeneration, a secreted *GFP *marker was developed for use in the present study. The presence of the secreted *GFP *was monitored at nearly all developmental stages (Figure 5) of the regeneration and was detectable as early as 5-7 days post-infection and reached maximum expression within 2-3 weeks. Strong expression of the secreted *GFP *in leaf guard cells (Figure 5E) was most commonly used to detect transgenic shoots. As soon as a shoot was confirmed as a transgenic, via detection of the secreted GFP, the shoot was transferred to media lacking hygromycin to further reduce the risk of hyperhydration.

### 6. Grafting technique to recover transgenic shoots into mature plants

Despite generating healthy elongated transgenic shoots (Figure 4B) we routinely failed to regenerate roots from transformed shoots, similar to reports in previous studies [7-9]. In most of the cases on rooting media, callus formation was observed from the base of the shoot yet no root formation was observed even after 4-6 weeks of culture with different hormone regimes. Our failures to generate rooted safflower plants forced the development of a grafting procedure to rescue transgenic shoots to allow their development into mature genetically-modified safflower plants.

**Figure 6:**
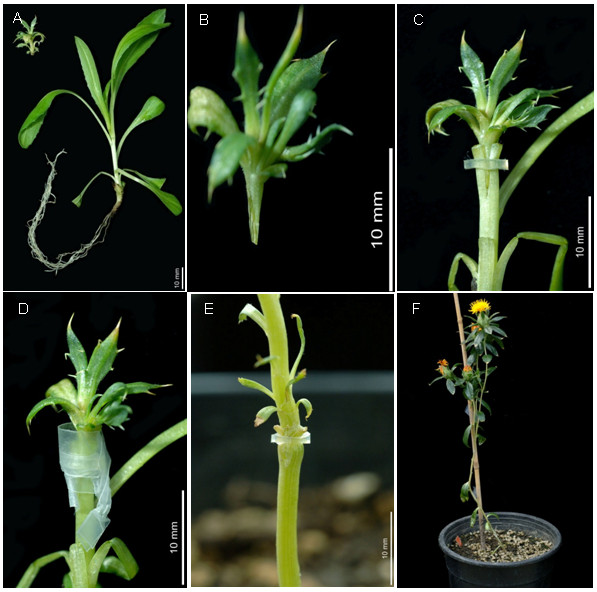
**Grafting of transgenic shoots on to seedling rootstocks of safflower**. (A) Rootstock seedling with well established root system and the transgenic scion, upper left corner. (B) V-shaped transgenic scion to be grafted onto a prepared seedling rootstock. (C) Rubber ring holding the scion and rootstock. (D) Graft union protected by parafilm. (E) Well established graft union. (F) Grafted safflower plant during seed development with multiple flower heads.

Scions isolated from elongated shoots were grafted onto rootstocks to overcome the poor *de novo *root formation in safflower (Figure 6). When scions were 1.5-2.0 cm long and rootstocks three weeks old, our grafting protocol resulted in approximately 50% success rate as assessed by their continued growth into mature safflower plants (Figure 6). Unsuccessful grafts were obvious after 3-5 days, whilst successful grafts flourished and new leaves were formed within two weeks. The most critical factor for a successful grafting was the length of the scion and age of the rootstock. Scions of 0.5-1 cm long had a lower survival rate (15 to 20%) than larger ones with size of 1.0-2 cm (45 to 50%). Similarly, high survival rates were reported with larger scions (0.6-1.5 cm) in grafted cotton [15,27]. Small scions became dehydrated despite the presence of the silicone tube and parafilm around the graft point. With smaller scions it was also difficult to make good contact between the scion and rootstock. The effect of the rootstock genotype on the survival of grafts was also evaluated however the overall scion and rootstock varietal differences were statistically not significant (Table 2). In our glasshouse conditions grafted safflower plants matured after 3 months and produced between 2 and 5 flower heads (Figure 6 F), each head bearing between 5 and 30 T_1 _seeds.

**Table 2:** Effect of rootstock genotype on the survival of grafted S-317 safflower shoots

Rootstock Genotype^a^	Number of shoots grafted ^b^	Mean number of shoots survived per treatment ^c ^± S.E	Percentage of success
WT	16	6.6 ± 0.3 ^d^	39%
S-317	16	8.3 ± 0.6 ^d^	52%

^a ^3 week old root stock;

^b ^Scion size approximately 1.5-2.0 cm

^c ^Graft survival measured as new leaves formed within 3 weeks of grafting

^d ^Each treatment has 16 scions with 3 replications. Means were compared using the Multiple Pair wise Comparison Procedure (Holm-Sidak method) and found to be not significantly different.

The survival of transgenic shoots and plants throughout the entire transformation protocol is presented in Table 3, a data set generated from 20 different infections that produced 110 mature GM safflower plants. A combination of improvements were required to generate healthy and elongated *GFP *positive shoots, including optimised T-DNA transfer conditions and reduced hyperhydricity and necrosis. Grafting was, however, absolutely required to rescue GM-shoots to allow their development into mature plants. Grafting is a reasonably robust method to recover these transgenic shoots, with an approximate 40 to 50% success rate (Table 2 and 3). Notably, such young grafts always survived to develop into mature seed-setting plants, suggesting that our protocol has addressed many issues regarding the acclimatisation of safflower from tissue culture conditions to soil grown conditions.

**Table 3:** Survival of transgenic shoots and plants through critical steps of the transformation protocol

Genotype	Cotyledons co-cultivated with *Agrobacterium*^a^	Shoots ready to graft obtained^b^	Successful grafts^c^	Mature plants obtained^d^	Final transformation efficiency^e^
S-317	1206	115	58	58	4.8%
WT	1660	95	52	52	3.1%
combined	2866	210	110	110	3.8%

^a ^Summary of a total of 20 independent experiments

^b ^Shoots are positive for *GFP *and at least 3 cm in length

^c ^Successful grafts as judged by new leaf growth within 2 weeks of grafting, yet still growing in controlled tissue culture conditions

^d ^Mature plants grown in soil and in a glasshouse

^e ^Transformation efficiency calculated as the (mature plants/infected cotyledons)x100

### 7. Molecular characterization of transgenic plants

**Figure 7:**
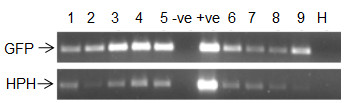
**PCR amplification of transgenes from T_0 _safflower lines**. Lanes 1-5 are PCR products generated from five genetically-modified S-317 lines, and lanes 8-11 are generated from four genetically-modified WT lines. -ve = Negative control non-transformed lines of S-317. +ve = Positive control (plasmid DNA from pCW265), H = sterile distilled water.

**Figure 8:**
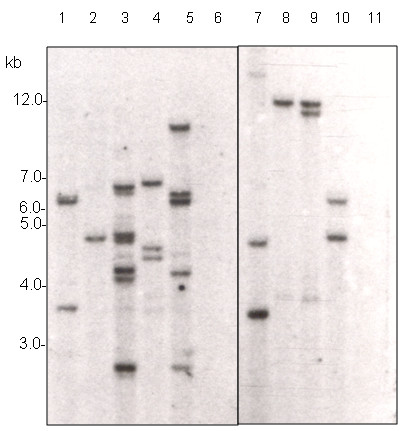
**Southern-blot analysis of nine independent lines of transgenic safflower**. Genomic DNA was prepared from the leaves of independent T_0 _lines from two varieties; S317 or WT. gDNA was digested with the restriction enzyme *HindIII *and probed using a labeled *GFP *sequence. Lane 1-5 transgenic plants from S-317 and lane 7-10 transgenic plants from WT. Lanes 6 and 11 are non-transformed S-317 and WT plants, respectively

**Figure 9:**
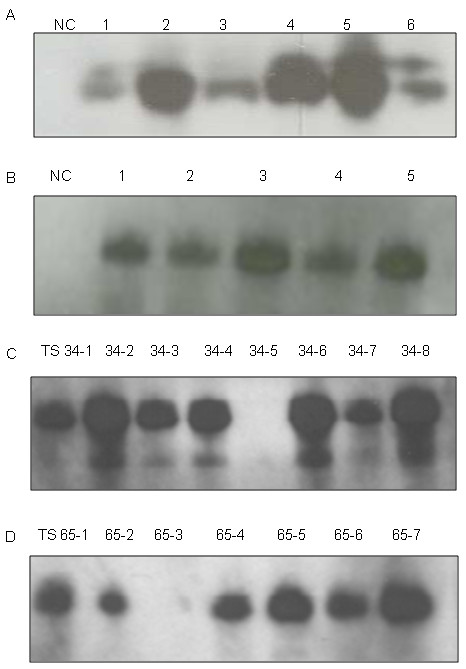
**Western blot analysis of *GFP *expression in T_0 _and T_1 _progeny of transgenic safflower plants**. (A) Lane 2-7 T_0 _plants from WT genotype. (B) Lane 2-6 T_0 _plants from S-317 genotype, Non-transgenic safflower were used as a negative control (NC). (C) Lane1-8 T_1 _transgenic line TS-34 and randomly selected progeny (WT genotype). (D) Lane 1-7 transgenic line TS-65 and randomly selected progeny (S-317 genotype).

We report here the generation of 110 grafted T_0 _plants, each scored as positive for a *GFP *signal, and the presence of T-DNA sequences tested by PCR amplification on genomic DNA, and a sample of these are shown in Figure 7. The analysis of PCR amplification products revealed in all cases the presence of DNA encoding both the secreted *GFP *and hygromycin resistance genes in all transgenic lines and its absence in the untransformed negative control, indicating the selection procedure with hygromycin and *GFP *were optimal. Southern blot analysis was used to determine to further confirm T-DNA integration into T_0 _lines and also as an estimate of the number of T-DNA insertions per line. Total genomic DNA was digested with *HindIII*, an enzyme that cleaves only once in the T-DNA region and hybridized with a radioactive *GFP *probe. Different integration patterns of T-DNA and insertion numbers were found in T_0 _plants indicating independent insertion events had been generated. The number of T-DNA insertions ranged from 1-7 and a sample of these patterns is shown in Figure 8. The *GFP *expression was further confirmed via Western blot on 11 T_0 _transgenic plants (Figure 9A and 9B), all showing the expected ~30 kDa protein band. In contrast, no corresponding bands were detected in the negative control plants.

A key omission in all safflower protocols published to date is the lack of evidence for the production of stably-transformed progeny with Mendelian inheritance patterns. To confirm that the transgenes were transmitted to the next generation, T_0 _plants were grown to maturity, self pollinated to produce T_1 _seeds, and progeny grown to produce T_1 _plants. Siblings from 6 independent T_1 _lines, each line containing single copy T-DNA insertion patterns as determined by Southern blot analysis, were scored for *GFP *expression using either Western blot analysis (Figure 9C and 9D) or epifluorescence microscopy analysis (Table 4). In our limited sampling of two lines of T_1 _plants (TS34 and TS65) by Western blot the expected ~30 kDa protein band of *GFP *was observed in most progeny and with *GFP *negatives presumably due to null segregating siblings. Fluorescent microscopy allowed more progeny to be screened in a further four lines (TS72, TS57, TS82 and TS88) and each line displayed the transgene inheritance ratios of 3:1 (ratio of *GFP *positive to negative) that may be expected for simple Mendelian inheritance patterns of a dominant marker (Table 4).

**Table 4:** Inheritance patterns of *GFP *in T_1 _progeny of four transgenic safflower lines

Transgenic line	Observed ^a^		Expected ^b^		Chi-square	P value
	*GFP*+	*GFP*-	*GFP*+	*GFP*-		
WT-TS-72	13	3	12	4	0.330	0.563
S-317-TS-57	17	7	18	6	0.220	0.637
S-317-TS-82	18	4	16.5	5.5	0.545	0.460
S-317-TS-88	33	13	32.5	13.5	0.026	0.871

^a ^*GFP *expression was scored using fluorescence microscopy on germinating seed.

^b ^Expected ratio if a dominant trait at a single locus is inherited by progeny (3:1 segregation ratio).

These molecular analyses confirm that grafted transgenic T_0 _safflower plants produce transgenic T_1 _safflower progeny that display classic inheritance patterns. Our method has so far produced over 110 mature genetically modified safflower plants, and the progeny of each of these lines have been screened as positive for *GFP *fluorescence, confirming their transgenic nature. Southern blot analysis also indicated that this transformation scheme produces relatively simple T-DNA insertion events in safflower, a feature that becomes important in gene regulatory procedures. Overall these results represent the first evidence for a method to reliably generate transgenic safflower seed.

## Conclusions

**Figure 10:**
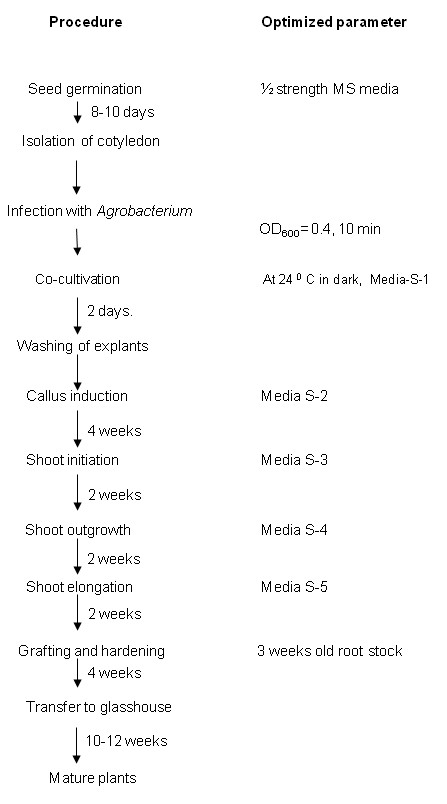
**Schematic representation of the complete protocol for the generation of GM safflower**.

Here we have developed an efficient genetic transformation method for both high oleic and high linoleic safflower varieties and the overall scheme is presented in Figure 10. We have made improvements in the transformation vector, *Agrobacterium *pre-treatments and regeneration conditions that allow the streamlined production of transgenic shoots. Despite these improvements the development of a grafting method to overcome the poor *de novo *rooting was critical for the production of mature GM safflower plants that produce GM safflower progeny. This protocol can now allow safflower to expand as an industrial crop platform and also offers the chance to modernise this ancient crop to benefit from advances in plant molecular biology.

## Methods

### Plant material

Seeds of safflower *(Carthamus tinctorius L.) *cultivars S-317 (supplied by Devexco International; high oleic content seed ~70% oleic acid) and a non-commercial variety WT (high linoleic type ~ 70% linoleic acid) were used in the present study. Seeds were soaked in warm (40°C) 10% Decon90 (Decon Laboratories Ltd.) for 30 min with shaking. After discarding the detergent solution, Carbendzim 2% w/v (Sigma) was added and agitated for 30 min and subsequently rinsed with sterile distilled water. Further sterilization was carried out with 0.2% HgCl_2 _solution for 15 min. Traces of HgCl_2 _were removed by several rinses with sterile distilled water. Sterilized seeds were germinated aseptically on half-strength MS media in tissue culture pots (200 ml) and incubated at 24°C in semi-light (50-100 Lux) for 6-8 days. Excised cotyledons from 6-8 day old seedlings were used in the present study.

### Bacterial strains and plasmids

The *npt-II *resistance gene from pORE3 [19] was removed using *FseI and AscI *digestion and replaced with a PCR amplified version of the intron-interrupted hygromycin resistance gene of the pVEC8 vector series [20] creating pORE6. The coding region of the secreted *GFP *from pCW141 [28] was PCR amplified to generate an entry clone flanked by *AttL *sites, pCW227, and a recombinase reaction used to introduce this into a compatible binary vector, pCW001, under the control of the *CaMV35S *promoter and ocs polyadenylation signal, producing pCW229. The entire 35Sp-SecretedGFP-ocs region was PCR amplified and introduced into the *SfoI *site of pORE6 to create pCW265. Thus-pCW265 is a T-DNA binary vector with hygromycin as a plant selectable marker, a secreted *GFP *to provide a non-toxic visual selectable marker and 13 unique restriction sites to allow the insertion of new sequences of interest (Figure 1). The multiple cloning site, including the *HindIII *site used for southern digest analysis, lies 2700 bp from the left border and 3400 bp from the right border. pCW265 was transformed into *Agrobacterium tumefaciens *strain AGL1 [29] via electroporation.

### Transformation and plant regeneration

#### Preparation of *Agrobacterium *and explant co-cultivation

Three days prior to transformation, 25 μl of an *Agrobacterium *AGL1 cells harboring plasmid pCW265 from -80°C was added to 25 ml of solid LB media with kanamycin 50 mg l^-1 ^and rifampcin 25 mg l^-1 ^and grown for two days at 28°C. One full loop (3 mm) bacteria culture was scraped from a 2-day old plate and suspended in 10 ml liquid LB with kanamycin 50 mg l^-1 ^and rifampcin 25 mg l^-1 ^and grown overnight at 28°C with agitation at 150 rpm. The bacterial cells were pelleted, re-suspended in 10 ml of Winans' AB medium (pH 5.8) and re-grown with kanamycin 50 mg l^-1 ^and rifampcin 25 mg l^-1 ^for 16 hrs with 100 μM AS. Such cultures were adjusted to OD_600 nm _= 0.4 and 1 mM SPR added for the final 2 hours of growth before explant co-cultivation. Freshly isolated cotyledons (~25) were infected with 10 ml *Agrobacterium *culture for 10 min, including a gentle inversion every 2 minutes during the infection. Explants infected with *Agrobacterium *were blotted on sterile filter paper to remove excess *Agrobacterium *and transferred to co-cultivation media (Table 1). All the plates were sealed with parafilm and incubated in the dark at 23-24°C for 48 hrs.

### Selection and plant regeneration

Two days after *Agrobacterium *infection explants were washed with sterile distilled water containing 500 mg l^-1 ^cefotaxime and 50 mg l^-1 ^timentin for 10 min and further rinsed in sterile distilled water for 10 min to remove the antibiotics and then blot dried on sterile filter paper and transferred onto petri dishes (90 mm in diameter) containing 25 ml of selection media (S2, Table 1). Un-inoculated explants were cultured in the same way as explants inoculated with *Agrobacterium *as a routine control experiment. After 8-10 weeks, hygromycin resistant and GFP positive shoots were excised and cultured on shoot elongation media. Regenerated shoots exhibiting green fluorescence were considered putatively transgenic; shoots showing red chlorophyll auto-inflorescence were designated as escapes and discarded.

### Recovery of transgenic plants

Plants destined to become rootstocks were sterilized as described above. Healthy seedlings were selected and transferred to prepared plastic bags (10 × 6 cm) containing seedling raising mix (50% perlite/vermiculite, 30% river sand and 20% top soil) with added fertilizer (1 g l^-1^) Aboska brand: N (14.2%), P (6.4%), K (5%) and CaCO_3 _(2 g l^-1^) and grown at 24°C in 16/8 hr (day/night) photoperiod for another 2 weeks until ready for grafting. Such plastic bags were found to be ideal for a reduced fungal contaminant of seedlings and subsequent transfer into pots at later stages.

Suitably elongated shoots (~3 cm long and with 2-3 true leaves) were collected and used for grafting (Figure 6A). Just prior to grafting, rootstock seedlings were decapitated (Additional file 3), with roots still in soil, by a single horizontal slice through the stem approximately 6-8 cm above the cotyledonary node. A second vertical cut 3-5 mm deep was made with a sterile micro-scalpel and a silicone tube (3-4 mm internal diameter) placed over the rootstock. The base of the suitable scion was cut to prepare a matching v-shape (Figure 6B) and inserted in the prepared rootstock. The silicon ring was moved up and adjusted to hold both scion and root stock (Figure 6C) and the graft point was wrapped with parafilm (Figure 6 D). Grafted plantlets were covered with 250 ml tissue culture pots to prevent desiccation (Additional file 3), and grown at 24°C with 2000 lux 16/8 hr (day/night) photoperiod for 1-2 weeks or until 3-4 new leaves had grown from the scion. At this point the covering pots were removed from successful grafts and plants were grown in the same conditions for a further two weeks. Unsuccessful grafts were discarded. Grafted plantlets with 5-6 new leaves were transferred to bigger pots (25 cm) with seedling raising mix and grown in a glasshouse facility in Canberra, ACT, until maturity, usually requiring 3-4 months. The procedure of transferring the grafted seedling from the plastic bag to a larger pot (25 cm) is also shown in Additional file 3, highlighting the excellent growth of roots from the rootstocks and the placement of the grafted plants into the soil.

### Detection of *GFP *via microscopy

*GFP *expression in putative transformed shoots grown under selective conditions was observed with a Leica dissecting microscope equipped with an epifluorescence attachment. Two different sets of filters were used: *GFP*2- a 510 nm long pass emission filter (transmitting red and green light) with a 480/40 nm excitation filter and *GFP*3- a 525/50 nm emission filter (transmitting only green light) with a 470/40 nm excitation filter.

### Experimental design and data analysis

All treatments were calculated as the mean and standard error (SE) of at least three replicates with a total data points typically with n = 30 except where indicated in the text. All treatments were compared using the Holm-Sidak test for Multiple Pairwise Comparisons in Sigma Plot (Version 11) using a level of significance (α) set at 0.05. Treatments considered to be not significantly different are marked with the same letter within the appropriate subgroup of comparisons (WT or S317 genotype).

### DNA extraction and PCR

Total genomic DNA was isolated from *GFP*-positive and hygromycin resistant young leaf tissue using the caesium chloride gradient method as described by [31]. PCR amplification was carried out to confirm genomic integration of the *GFP *and *hph *genes using either *GFP*-specific primer pairs GFP-F 5' ACACCCTGGTGAACCGCATCG and GFP-R 5' CGGCGGTCACGAACTCCAGC and HPH-F 5' AAAAGCCTGAACTCACCGC and HPH-R 5' TCGTCCATCACAGTTTGCC. The thermal profile of the PCR was: initial denaturation at 94°C for 15 min, 35 cycles of 92°C for 30s, 52°C for 30s, 72°C for 1 min, and finally 72°C for 10 min. Amplified products were size fractionated on 0.8% w/v agarose gel in TAE buffer. Gel electrophoresis was carried out at 80 volts for 40 min before DNA bands were visualized with a BioRad QuantiOne UV transilluminator and software.

### Southern blot analysis

Total genomic DNA was isolated from transgenic young leaves using caesium chloride method following the standard procedure of [32]. 5 μg of DNA was digested with *HindIII*, electrophoresed through 1.0% agarose gel and DNA transferred onto Hybond N^+ ^membranes (Amersham, UK). A purified 966 bp DNA fragment containing the *GFP *gene was excised from pCW229 by digestion with *NotI *and was ^32^P-labelled using the Megaprime DNA labeling system (Amersham) and used as a probe.

### Westerns blot analysis

*GFP *was detected in extracts of safflower leaves using an earlier reported protocol [30]. Briefly, 100 mg fresh safflower leaf was crushed in 200 μL laemelli buffer (2% SDS, 100 mM Tris-base, pH 7.2, 20% glycerol, 60 mM dithiothreol; 0.02% bromophenol blue) and heated at 95°C for 5 min before centrifuging at room temperature at 13000 g for 5 min. 10 μL of supernatant was loaded onto a 12.5% polyacrylamide gel (12.5% PAGE-Sprint Buffer System; Amresco laboratory supplies) and resolved using 200 volts for 60 min. Fermentas Page Ruler Plus was used as an indicator of protein size standards. Protein gels were semi-dry electroblotted at 150 mA for 2 hours onto PVDF membranes (Sigma) and probed for *GFP *using an anti-*GFP *monoclonal primary antibody (1/5000 dilution, Clontech), a goat anti-mouse HRP-secondary antibody (1/5000 dilution, Promega) and enhanced chemical luminescence (Lightning ECL, Amersham).

## Abbreviations

TDZ: Thidiazuron; BA: Benzylamineo purine; 2iP: 6-(c,c-Dimethylallylamino) purine; NAA -α: Naphthalene acetic acid; GFP: Green Fluorescent Protein; Hph: Hygromycin; SPR: Spermidine; AS: Acetosyringone; MS: Murashige and Skoog media; PVP: Polyvinylpyrrolidone; AgNO_3_: Silver nitrate; KN: Kinetin/6-furfurylaminopurine.

## Competing interests

The authors declare that they have no competing interests.

## Authors' contributions

BS, SPS, AGG and CCW designed the experiments. BS and LH performed all the tissue culture and transformation experiments. LH did the grafting and Southern blot analysis. CCW constructed the vector pCW265 and performed Western blot analysis. BS and CW wrote the manuscript. All authors read and approved the final manuscript.

## Supplementary Material

Additional file 1 **Differences in the appearance of transformed shoots with cytoplasmic *GFP *and a secreted *GFP *in safflower**. (A) Cytoplasmic *GFP *(B) Secreted *GFP*. Note that although the fluorescence is much brighter in cytoplasmic versus the secreted *GFP*, the cytoplasmic *GFP *causes tissue swelling before death. Click here for file

Additional file 2 **Localisation of the secreted *GFP *in safflower**. (A) *GFP *expression in transgenic leaf tissue where each pavement cell is lined with a GFP signal. (B) *GFP *expression in individual cells of calli that are typically rounder than those in leaf cells in Panel A. Click here for file

Additional file 3 **Grafting of transgenic shoots and their transfer to larger pots**. (A) Decapitated seedling (roots are still in the soil). (B) An example of a grafted shoot covered with plastic container to maintain the humidity for 2 weeks. (C) Removal of grafted seedling from plastic bag without disturbing the root. (D-E) Gentle transfer of grafted seedling to the pot. Click here for file
