# Supplementary figures and images for: Agrobacterium-mediated transformation of safflower and the efficient recovery of transgenic plants via grafting
Source: Plant Methods. 2011 May 20;7:12. doi: 10.1186/1746-4811-7-12 (PMC3115923; doi:10.1186/1746-4811-7-12)

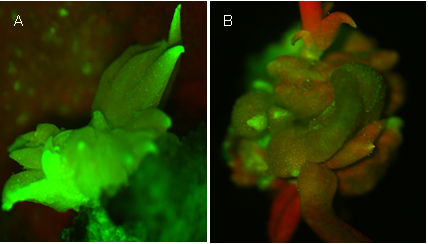

Supplement: Additional file 1 — Differences in the appearance of transformed shoots with cytoplasmic GFP and a secreted GFP in safflower. (A) Cytoplasmic GFP (B) Secreted GFP. Note that although the fluorescence is much brighter in cytoplasmic versus the secreted GFP, the cytoplasmic GFP causes tissue swelling before death. [file 1746-4811-7-12-S1.TIFF]

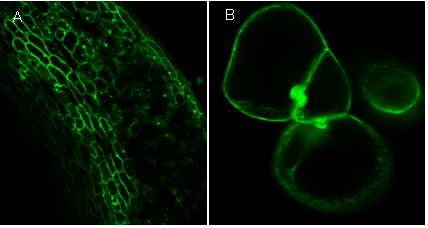

Supplement: Additional file 2 — Localisation of the secreted GFP in safflower. (A) GFP expression in transgenic leaf tissue where each pavement cell is lined with a GFP signal. (B) GFP expression in individual cells of calli that are typically rounder than those in leaf cells in Panel A. [file 1746-4811-7-12-S2.TIFF]

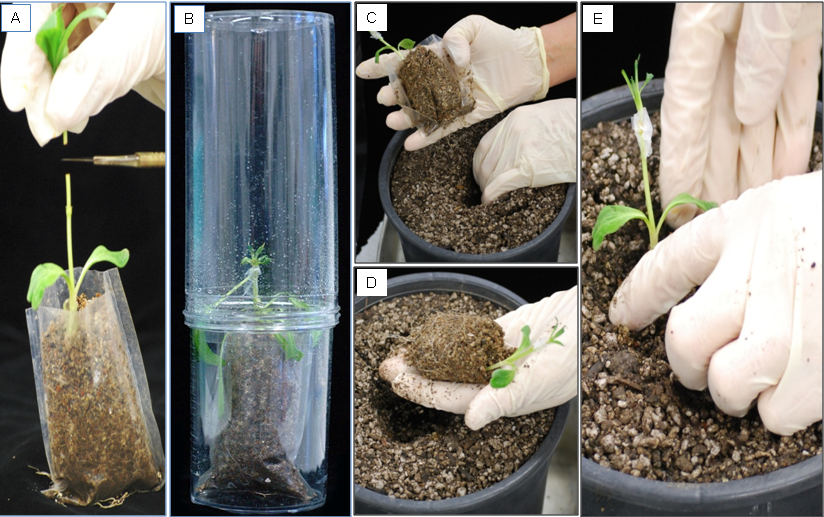

Supplement: Additional file 3 — Grafting of transgenic shoots and their transfer to larger pots. (A) Decapitated seedling (roots are still in the soil). (B) An example of a grafted shoot covered with plastic container to maintain the humidity for 2 weeks. (C) Removal of grafted seedling from plastic bag without disturbing the root. (D-E) Gentle transfer of grafted seedling to the pot. [file 1746-4811-7-12-S3.TIFF]
